# Supplementary material for: Results of two cross-sectional database analyses regarding nap-induced modulations of tinnitus
Source: Sci Rep. 2024 Aug 29;14:20111. doi: 10.1038/s41598-024-70871-z (PMC11362562; doi:10.1038/s41598-024-70871-z)
Supplement: Supplementary file 1 — Supplementary Table 1. [file 41598_2024_70871_MOESM1_ESM.docx]

**Supplementary Table 1**. Extended group comparison in the TRI database. *SD : Standard Deviation, TW : Tinnitus worsens after naps, NE : No effect of naps on tinnitus, TI : Tinnitus improves after naps, THI : Tinnitus Handicap Inventory, TQ : Tinnitus Questionnaire, WHOQOL : The World Health Organization Quality of Life, * : p < 0.05 before Holm correction, ** : p < 0.05 after Holm correction.*

|  | **Worsens N= 642** | **No effect N= 2651** | **Improves N= 334** | **Statistic** | **p -value** | **Effect size** |  | **Post-hoc test** | | **Post-hoc effect size** |
| --- | --- | --- | --- | --- | --- | --- | --- | --- | --- | --- |
| **Age (years)** |  |  |  | H = 13.2** | 0.04 | 0.003 (Negligible) |  | |  |  |
| Mean (SD) | 61.8 (14.67) | 59.4 (13.45) | 59.7 (14.6) |  |  |  | TW VS NE | | 0.012 | 0.175 (Negligible) |
| Median [Min Max] | 61.5 [21, 102] | 60 [18, 103] | 60 [21, 100] |  |  |  | TI VS NE | | 1.0 | 0.024 (Negligible) |
| Missing | 0 (0%) | 2 (0.1%) | 0 (0%) |  |  |  | TW VS TI | | 0.52 | 0.141 (Negligible) |
| **Tinnitus duration (months)** |  |  |  | H = 2.2 | 1.0 | 0.0 (Negligible) |  | |  |  |
| Mean (SD) | 103.9 (111.89) | 100.9 (112.93) | 93.4 (102.16) |  |  |  |  | |  |  |
| Median [Min Max] | 55 [0.8, 604] | 53 [0.5, 726] | 49.1 [0.6, 580] |  |  |  |  | |  |  |
| Missing | 70 (10.9%) | 270 (10.2%) | 33 (9.9%) |  |  |  |  | |  |  |
| **Tinnitus pitch (0: very high frequency, 1: high, 2: medium, 3: low frequency)** |  |  |  | H = 8.3* | 0.248 | 0.002 (Negligible) |  | |  |  |
| Mean (SD) | 0.9 (0.72) | 1 (0.74) | 1 (0.77) |  |  |  |  | |  |  |
| Median [Min Max] | 1 [0, 3] | 1 [0, 3] | 1 [0, 3] |  |  |  |  | |  |  |
| Missing | 35 (5.5%) | 84 (3.2%) | 11 (3.3%) |  |  |  |  | |  |  |
| **Tinnitus laterality (0 : bilateral, 0.5 : More on one side, 1 : unilateral)** |  |  |  | H = 6.5* | 0.541 | 0.001 (Negligible) |  | |  |  |
| Mean (SD) | 0.5 (0.36) | 0.5 (0.4) | 0.5 (0.4) |  |  |  |  | |  |  |
| Median [Min Max] | 0.5 [0, 1] | 0.5 [0, 1] | 0.5 [0, 1] |  |  |  |  | |  |  |
| Missing | 5 (0.8%) | 13 (0.5%) | 1 (0.3%) |  |  |  |  | |  |  |
| **Average loudness (0-100)** |  |  |  | H = 13.5** | 0.035 | 0.003 (Negligible) |  | |  |  |
| Mean (SD) | 66.6 (22.57) | 64.4 (22.63) | 61.1 (23.43) |  |  |  | TW VS NE | | 0.52 | 0.098 (Negligible) |
| Median [Min Max] | 70 [0, 100] | 70 [0, 100] | 60 [0, 100] |  |  |  | TI VS NE | | 0.305 | 0.145 (Negligible) |
| Missing | 27 (4.2%) | 85 (3.2%) | 7 (2.1%) |  |  |  | TW VS TI | | 0.01 | 0.24 (Small) |
| **THI score (0-100)** |  |  |  | H = 53.8** | p < 0.001 | 0.015 (Small) |  | |  |  |
| Mean (SD) | 53.6 (22.29) | 46.8 (23.33) | 52.2 (21.75) |  |  |  | TW VS NE | | p < 0.001 | 0.297 (Small) |
| Median [Min Max] | 54 [0, 100] | 46 [0, 100] | 50 [4, 100] |  |  |  | TI VS NE | | 0.002 | 0.236 (Small) |
| Missing | 13 (2%) | 41 (1.5%) | 6 (1.8%) |  |  |  | TW VS TI | | 1.0 | 0.064 (Negligible) |
| **TQ score (0-84)** |  |  |  | H = 23.8** | p < 0.001 | 0.008 (Negligible) |  | |  |  |
| Mean (SD) | 43.9 (17.19) | 39.7 (17.91) | 41.5 (16.76) |  |  |  | TW VS NE | | p < 0.001 | 0.233 (Small) |
| Median [Min Max] | 44 [4, 84] | 39 [1, 84] | 41 [4, 79] |  |  |  | TI VS NE | | 1.0 | 0.099 (Negligible) |
| Missing | 130 (20.2%) | 612 (23.1%) | 75 (22.5%) |  |  |  | TW VS TI | | 1.0 | 0.14 (Negligible) |
| **How much of a problem is your tinnitus at present? (0 : no problem, 5 : a very big problem** |  |  |  | H = 18.4** | 0.004 | 0.005 (Negligible) |  | |  |  |
| Mean (SD) | 2.6 (0.9) | 2.4 (0.89) | 2.4 (0.85) |  |  |  | TW VS NE | | 0.001 | 0.193 (Negligible) |
| Median [Min Max] | 3 [0, 4] | 2 [0, 4] | 2 [0, 4] |  |  |  | TI VS NE | | 1.0 | 0.014 (Negligible) |
| Missing | 53 (8.3%) | 187 (7.1%) | 35 (10.5%) |  |  |  | TW VS TI | | 0.098 | 0.209 (Small) |
| **How STRONG or LOUD is your tinnitus at present? (0 to 10 scale)** |  |  |  | H = 15.2** | 0.017 | 0.004 (Negligible) |  | |  |  |
| Mean (SD) | 6.7 (2.15) | 6.4 (2.19) | 6.1 (2.23) |  |  |  | TW VS NE | | 0.353 | 0.108 (Negligible) |
| Median [Min Max] | 7 [0, 10] | 7 [0, 10] | 6 [0, 10] |  |  |  | TI VS NE | | 0.235 | 0.154 (Negligible) |
| Missing | 34 (5.3%) | 98 (3.7%) | 24 (7.2%) |  |  |  | TW VS TI | | 0.005 | 0.263 (Small) |
| **How UNCOMFORTABLE is your tinnitus at present, if everything around you is quiet? (0 to 10 scale)** |  |  |  | H = 27.9** | p < 0.001 | 0.007 (Negligible) |  | |  |  |
| Mean (SD) | 7.2 (2.23) | 7.1 (2.33) | 6.4 (2.47) |  |  |  | TW VS NE | | 1.0 | 0.059 (Negligible) |
| Median [Min Max] | 8 [0, 10] | 8 [0, 10] | 7 [1, 10] |  |  |  | TI VS NE | | p < 0.001 | 0.303 (Small) |
| Missing | 33 (5.1%) | 104 (3.9%) | 25 (7.5%) |  |  |  | TW VS TI | | p < 0.001 | 0.367 (Small) |
| **How ANNOYING is your tinnitus at present? (0 to 10 scale)** |  |  |  | H = 10.6* | 0.111 | 0.002 (Negligible) |  | |  |  |
| Mean (SD) | 6.9 (2.41) | 6.8 (2.44) | 6.4 (2.42) |  |  |  |  | |  |  |
| Median [Min Max] | 8 [0, 10] | 7 [0, 10] | 7 [0, 10] |  |  |  |  | |  |  |
| Missing | 31 (4.8%) | 97 (3.7%) | 24 (7.2%) |  |  |  |  | |  |  |
| **How easy is it for you to IGNORE your tinnitus at present? (0 to 10 scale)** |  |  |  | H = 14.1** | 0.026 | 0.003 (Negligible) |  | |  |  |
| Mean (SD) | 7.1 (2.51) | 6.9 (2.69) | 6.4 (2.71) |  |  |  | TW VS NE | | 1.0 | 0.082 (Negligible) |
| Median [Min Max] | 8 [0, 10] | 7 [0, 10] | 7 [0, 10] |  |  |  | TI VS NE | | 0.044 | 0.185 (Negligible) |
| Missing | 30 (4.7%) | 98 (3.7%) | 24 (7.2%) |  |  |  | TW VS TI | | 0.008 | 0.277 (Small) |
| **How UNPLEASANT is your tinnitus at present? (0 to 10 scale)** |  |  |  | H = 12.6* | 0.051 | 0.003 (Negligible) |  | |  |  |
| Mean (SD) | 7 (2.35) | 6.8 (2.44) | 6.4 (2.41) |  |  |  |  | |  |  |
| Median [Min Max] | 8 [0, 10] | 7 [0, 10] | 7 [0, 10] |  |  |  |  | |  |  |
| Missing | 30 (4.7%) | 94 (3.5%) | 23 (6.9%) |  |  |  |  | |  |  |
| **% of time aware of tinnitus (0-100)** |  |  |  | H = 2.0 | 1.0 | -0.0 (Negligible) |  | |  |  |
| Mean (SD) | 73.8 (25.31) | 71.1 (27.98) | 70.7 (27.56) |  |  |  |  | |  |  |
| Median [Min Max] | 80 [5, 100] | 80 [0, 100] | 80 [1, 100] |  |  |  |  | |  |  |
| Missing | 12 (1.9%) | 20 (0.8%) | 6 (1.8%) |  |  |  |  | |  |  |
| **% of time annoyed, distressed, irritated by your tinnitus (0-100)** |  |  |  | H = 10.6* | 0.111 | 0.002 (Negligible) |  | |  |  |
| Mean (SD) | 61.5 (29.45) | 56.9 (31.14) | 59.3 (30.77) |  |  |  |  | |  |  |
| Median [Min Max] | 70 [0, 100] | 50 [0, 100] | 60 [0, 100] |  |  |  |  | |  |  |
| Missing | 9 (1.4%) | 44 (1.7%) | 3 (0.9%) |  |  |  |  | |  |  |
| **WHOQOL : Physical Health (0-20)** |  |  |  | H = 9.5* | 0.154 | 0.003 (Negligible) |  | |  |  |
| Mean (SD) | 12.4 (1.86) | 12.7 (1.77) | 12.7 (1.75) |  |  |  |  | |  |  |
| Median [Min Max] | 13 [7, 18] | 13 [5, 18] | 13 [6, 17] |  |  |  |  | |  |  |
| Missing | 113 (17.6%) | 448 (16.9%) | 66 (19.8%) |  |  |  |  | |  |  |
| **WHOQOL : Psychological Health (0-20)** |  |  |  | H = 12.3* | 0.056 | 0.003 (Negligible) |  | |  |  |
| Mean (SD) | 13.4 (2.11) | 13.8 (2) | 13.9 (1.87) |  |  |  |  | |  |  |
| Median [Min Max] | 14 [7, 18] | 14 [6, 19] | 14 [7, 18] |  |  |  |  | |  |  |
| Missing | 110 (17.1%) | 448 (16.9%) | 65 (19.5%) |  |  |  |  | |  |  |
| **WHOQOL : Social Factors (0-20)** |  |  |  | H = 7.9* | 0.288 | 0.002 (Negligible) |  | |  |  |
| Mean (SD) | 14.4 (3.41) | 14.8 (3.27) | 14.4 (3.21) |  |  |  |  | |  |  |
| Median [Min Max] | 15 [4, 20] | 16 [4, 20] | 15 [5, 20] |  |  |  |  | |  |  |
| Missing | 111 (17.3%) | 449 (16.9%) | 67 (20.1%) |  |  |  |  | |  |  |
| **WHOQOL : Environmental Factors (0-20)** |  |  |  | H = 12.4* | 0.054 | 0.003 (Negligible) |  | |  |  |
| Mean (SD) | 16.3 (2.43) | 16.6 (2.18) | 16.3 (2.23) |  |  |  |  | |  |  |
| Median [Min Max] | 16.5 [6, 20] | 17 [6, 20] | 16 [10, 20] |  |  |  |  | |  |  |
| Missing | 112 (17.4%) | 437 (16.5%) | 64 (19.2%) |  |  |  |  | |  |  |
| **Degree of hyperacusis (0 : None, 4 : Very important)** |  |  |  | H = 25.5** | p < 0.001 | 0.007 (Negligible) |  | |  |  |
| Mean (SD) | 2.3 (1.15) | 2.2 (1.21) | 2.5 (1.2) |  |  |  | TW VS NE | | 1.0 | 0.088 (Negligible) |
| Median [Min Max] | 2 [0, 4] | 2 [0, 4] | 2 [0, 4] |  |  |  | TI VS NE | | p < 0.001 | 0.291 (Small) |
| Missing | 6 (0.9%) | 16 (0.6%) | 1 (0.3%) |  |  |  | TW VS TI | | 0.066 | 0.211 (Small) |
| **Gender** |  |  |  | Chi2 = 10.4* | 0.111 | 0.038 (Negligible) |  | |  |  |
| Male | 457 (71.2%) | 1726 (65.1%) | 209 (62.6%) |  |  |  |  | |  |  |
| Female | 185 (28.8%) | 925 (34.9%) | 125 (37.4%) |  |  |  |  | |  |  |
| Missing | 0 (0%) | 0 (0%) | 0 (0%) |  |  |  |  | |  |  |
| **Handedness** |  |  |  | Chi2 = 6.1 | 1.0 | 0.021 (Negligible) |  | |  |  |
| Right-handed | 517 (81.2%) | 2205 (83.8%) | 274 (82.3%) |  |  |  |  | |  |  |
| Ambidextrous | 47 (7.4%) | 181 (6.9%) | 18 (5.4%) |  |  |  |  | |  |  |
| Left-handed | 73 (11.5%) | 245 (9.3%) | 41 (12.3%) |  |  |  |  | |  |  |
| Missing | 5 (0.8%) | 20 (0.8%) | 1 (0.3%) |  |  |  |  | |  |  |
| **Hearing difficulties** |  |  |  | Chi2 = 6.1* | 0.617 | 0.029 (Negligible) |  | |  |  |
| Yes | 357 (56.8%) | 1597 (61.2%) | 212 (64.4%) |  |  |  |  | |  |  |
| No | 271 (43.2%) | 1013 (38.8%) | 117 (35.6%) |  |  |  |  | |  |  |
| Missing | 14 (2.2%) | 41 (1.5%) | 5 (1.5%) |  |  |  |  | |  |  |
| **Tinnitus type of sound** |  |  |  | Chi2 = 18.9* | 0.102 | 0.029 (Negligible) |  | |  |  |
| Tonal | 366 (58.6%) | 1728 (66.2%) | 192 (58.5%) |  |  |  |  | |  |  |
| Noise | 84 (13.4%) | 298 (11.4%) | 43 (13.1%) |  |  |  |  | |  |  |
| Criquets | 107 (17.1%) | 378 (14.5%) | 60 (18.3%) |  |  |  |  | |  |  |
| Other | 68 (10.9%) | 207 (7.9%) | 33 (10.1%) |  |  |  |  | |  |  |
| Missing | 17 (2.6%) | 40 (1.5%) | 6 (1.8%) |  |  |  |  | |  |  |
| Tinnitus sound pulsatile |  |  |  | Chi2 = 14.8* | 0.111 | 0.032 (Negligible) |  | |  |  |
| Pulsatile following heart beats | 76 (12.2%) | 257 (9.9%) | 42 (12.9%) |  |  |  |  | |  |  |
| Pulsatile but not following heart beats | 70 (11.3%) | 227 (8.7%) | 42 (12.9%) |  |  |  |  | |  |  |
| Not pulsatile | 475 (76.5%) | 2114 (81.4%) | 242 (74.2%) |  |  |  |  | |  |  |
| Missing | 21 (3.3%) | 53 (2%) | 8 (2.4%) |  |  |  |  | |  |  |
| **Tinnitus side** |  |  |  | Chi2 = 47.6** | p < 0.001 | 0.033 (Negligible) |  | |  |  |
| Right ear | 67 (10.5%) | 340 (12.9%) | 52 (15.6%) |  |  |  | TW VS NE | | p < 0.001 | 0.044 (Small) |
| Left ear | 77 (12.1%) | 449 (17%) | 61 (18.3%) |  |  |  | TI VS NE | | 1.0 | 0.014 (Negligible) |
| Both ears, worse in left | 167 (26.2%) | 553 (21%) | 67 (20.1%) |  |  |  | TW VS TI | | 0.086 | 0.03 (Negligible) |
| Both ears, worse in right | 126 (19.7%) | 428 (16.2%) | 56 (16.8%) |  |  |  |  | |  |  |
| Both ears, equally | 109 (17.1%) | 618 (23.4%) | 65 (19.5%) |  |  |  |  | |  |  |
| Inside the head | 91 (14.3%) | 250 (9.5%) | 32 (9.6%) |  |  |  |  | |  |  |
| Elsewhere | 1 (0.2%) | 1 (0%) | 0 (0%) |  |  |  |  | |  |  |
| Missing | 4 (0.6%) | 12 (0.5%) | 1 (0.3%) |  |  |  |  | |  |  |
| **Family history of tinnitus complaints** |  |  |  | Chi2 = 0.1 | 1.0 | 0.003 (Negligible) |  | |  |  |
| Yes | 148 (23.5%) | 621 (23.9%) | 77 (23.5%) |  |  |  |  | |  |  |
| No | 483 (76.5%) | 1976 (76.1%) | 251 (76.5%) |  |  |  |  | |  |  |
| Missing | 11 (1.7%) | 54 (2%) | 6 (1.8%) |  |  |  |  | |  |  |
| **Tinnitus onset** |  |  |  | Chi2 = 3.2 | 1.0 | 0.021 (Negligible) |  | |  |  |
| Gradual | 287 (46.8%) | 1273 (50.4%) | 150 (47.2%) |  |  |  |  | |  |  |
| Abrupt | 326 (53.2%) | 1255 (49.6%) | 168 (52.8%) |  |  |  |  | |  |  |
| Missing | 29 (4.5%) | 123 (4.6%) | 16 (4.8%) |  |  |  |  | |  |  |
| **Tinnitus cause : noise trauma** |  |  |  | Chi2 = 1.0 | 1.0 | 0.012 (Negligible) |  | |  |  |
| Yes | 526 (96%) | 2147 (96.8%) | 277 (96.2%) |  |  |  |  | |  |  |
| No | 22 (4%) | 71 (3.2%) | 11 (3.8%) |  |  |  |  | |  |  |
| Missing | 94 (14.6%) | 433 (16.3%) | 46 (13.8%) |  |  |  |  | |  |  |
| **Tinnitus cause : psychological (stress, anxiety, depression)** |  |  |  | Chi2 = 21.7** | p < 0.001 | 0.055 (Negligible) |  | |  |  |
| Yes | 257 (46.9%) | 1143 (51.5%) | 108 (37.5%) |  |  |  | TW VS NE | | 1.0 | 0.031 (Negligible) |
| No | 291 (53.1%) | 1075 (48.5%) | 180 (62.5%) |  |  |  | TI VS NE | | p < 0.001 | 0.073 (Negligible) |
| Missing | 94 (14.6%) | 433 (16.3%) | 46 (13.8%) |  |  |  | TW VS TI | | 0.305 | 0.042 (Negligible) |
| **Tinnitus cause : whiplash** |  |  |  | Chi2 = 11.3* | 0.083 | 0.04 (Negligible) |  | |  |  |
| Yes | 510 (93.1%) | 2129 (96%) | 268 (93.1%) |  |  |  |  | |  |  |
| No | 38 (6.9%) | 89 (4%) | 20 (6.9%) |  |  |  |  | |  |  |
| Missing | 94 (14.6%) | 433 (16.3%) | 46 (13.8%) |  |  |  |  | |  |  |
| **Tinnitus cause : head trauma** |  |  |  | Chi2 = 0.1 | 1.0 | 0.004 (Negligible) |  | |  |  |
| Yes | 535 (97.6%) | 2165 (97.6%) | 282 (97.9%) |  |  |  |  | |  |  |
| No | 13 (2.4%) | 53 (2.4%) | 6 (2.1%) |  |  |  |  | |  |  |
| Missing | 94 (14.6%) | 433 (16.3%) | 46 (13.8%) |  |  |  |  | |  |  |
| **Tinnitus cause : noise trauma** |  |  |  | Chi2 = 3.6 | 1.0 | 0.022 (Negligible) |  | |  |  |
| Yes | 464 (84.7%) | 1830 (82.5%) | 229 (79.5%) |  |  |  |  | |  |  |
| No | 84 (15.3%) | 388 (17.5%) | 59 (20.5%) |  |  |  |  | |  |  |
| Missing | 94 (14.6%) | 433 (16.3%) | 46 (13.8%) |  |  |  |  | |  |  |
| **Tinnitus cause : other** |  |  |  | Chi2 = 4.0 | 1.0 | 0.024 (Negligible) |  | |  |  |
| Yes | 280 (51.1%) | 1178 (53.1%) | 168 (58.3%) |  |  |  |  | |  |  |
| No | 268 (48.9%) | 1040 (46.9%) | 120 (41.7%) |  |  |  |  | |  |  |
| Missing | 94 (14.6%) | 433 (16.3%) | 46 (13.8%) |  |  |  |  | |  |  |
| **Tinnitus varies from day to day** |  |  |  | Chi2 = 136.9** | p < 0.001 | 0.137 (Small) |  | |  |  |
| Yes | 489 (77.1%) | 1485 (56.8%) | 264 (80%) |  |  |  | TW VS NE | | p < 0.001 | 0.155 (Small) |
| No | 145 (22.9%) | 1128 (43.2%) | 66 (20%) |  |  |  | TI VS NE | | p < 0.001 | 0.133 (Small) |
| Missing | 8 (1.2%) | 38 (1.4%) | 4 (1.2%) |  |  |  | TW VS TI | | 1.0 | 0.016 (Negligible) |
| **Tinnitus intermittent or continuous ?** |  |  |  | Chi2 = 38.9** | p < 0.001 | 0.073 (Small) |  | |  |  |
| Intermittent | 112 (17.8%) | 307 (11.7%) | 74 (22.4%) |  |  |  | TW VS NE | | 0.002 | 0.067 (Negligible) |
| Continuous | 517 (82.2%) | 2317 (88.3%) | 256 (77.6%) |  |  |  | TI VS NE | | p < 0.001 | 0.09 (Negligible) |
| Missing | 13 (2%) | 27 (1%) | 4 (1.2%) |  |  |  | TW VS TI | | 1.0 | 0.027 (Negligible) |
| **Influence of stress over tinnitus** |  |  |  | Chi2 = 84.2** | p < 0.001 | 0.076 (Small) |  | |  |  |
| Worsens | 477 (77.2%) | 1742 (67.5%) | 294 (89.6%) |  |  |  | TW VS NE | | p < 0.001 | 0.057 (Negligible) |
| Improves | 8 (1.3%) | 29 (1.1%) | 0 (0%) |  |  |  | TI VS NE | | p < 0.001 | 0.097 (Small) |
| No effect | 133 (21.5%) | 809 (31.4%) | 34 (10.4%) |  |  |  | TW VS TI | | p < 0.001 | 0.057 (Negligible) |
| Missing | 24 (3.7%) | 71 (2.7%) | 6 (1.8%) |  |  |  |  | |  |  |
| **Sleep at night and tinnitus during the day** |  |  |  | Chi2 = 399.0** | p < 0.001 | 0.235 (Medium) |  | |  |  |
| Linked | 261 (71.5%) | 338 (22.8%) | 127 (68.6%) |  |  |  | TW VS NE | | p < 0.001 | 0.295 (Small) |
| Not linked | 104 (28.5%) | 1145 (77.2%) | 58 (31.4%) |  |  |  | TI VS NE | | p < 0.001 | 0.216 (Small) |
| Missing | 277 (43.1%) | 1168 (44.1%) | 149 (44.6%) |  |  |  | TW VS TI | | 1.0 | 0.01 (Negligible) |
| **Some sounds can worsen tinnitus** |  |  |  | Chi2 = 34.4** | p < 0.001 | 0.069 (Negligible) |  | |  |  |
| Yes | 374 (73.5%) | 1407 (66%) | 242 (81.2%) |  |  |  | TW VS NE | | 0.048 | 0.053 (Negligible) |
| No | 135 (26.5%) | 725 (34%) | 56 (18.8%) |  |  |  | TI VS NE | | p < 0.001 | 0.086 (Negligible) |
| Missing | 133 (20.7%) | 519 (19.6%) | 36 (10.8%) |  |  |  | TW VS TI | | 0.385 | 0.04 (Negligible) |
| **Some sounds can suppress tinnitus** |  |  |  | Chi2 = 15.5** | 0.015 | 0.046 (Negligible) |  | |  |  |
| Yes | 431 (78.9%) | 1665 (74.7%) | 238 (84.4%) |  |  |  | TW VS NE | | 0.859 | 0.033 (Negligible) |
| No | 115 (21.1%) | 564 (25.3%) | 44 (15.6%) |  |  |  | TI VS NE | | 0.016 | 0.058 (Negligible) |
| Missing | 96 (15%) | 422 (15.9%) | 52 (15.6%) |  |  |  | TW VS TI | | 1.0 | 0.03 (Negligible) |
| **Some sounds can cause physical discomfort** |  |  |  | Chi2 = 14.3** | 0.025 | 0.044 (Negligible) |  | |  |  |
| Yes | 359 (62.7%) | 1357 (58.9%) | 208 (69.8%) |  |  |  | TW VS NE | | 1.0 | 0.026 (Negligible) |
| No | 214 (37.3%) | 946 (41.1%) | 90 (30.2%) |  |  |  | TI VS NE | | 0.014 | 0.059 (Negligible) |
| Missing | 69 (10.7%) | 348 (13.1%) | 36 (10.8%) |  |  |  | TW VS TI | | 0.859 | 0.034 (Negligible) |
| **Somatosensory : Jaw or head movements can modulate tinnitus** |  |  |  | Chi2 = 98.6** | p < 0.001 | 0.117 (Small) |  | |  |  |
| Yes | 325 (51.5%) | 835 (32%) | 153 (46.6%) |  |  |  | TW VS NE | | p < 0.001 | 0.152 (Small) |
| No | 306 (48.5%) | 1777 (68%) | 175 (53.4%) |  |  |  | TI VS NE | | p < 0.001 | 0.087 (Negligible) |
| Missing | 11 (1.7%) | 39 (1.5%) | 6 (1.8%) |  |  |  | TW VS TI | | 1.0 | 0.023 (Negligible) |
| **Temporomandibular disorder** |  |  |  | Chi2 = 12.1* | 0.058 | 0.041 (Negligible) |  | |  |  |
| Yes | 176 (28%) | 646 (24.7%) | 109 (33.1%) |  |  |  |  | |  |  |
| No | 453 (72%) | 1966 (75.3%) | 220 (66.9%) |  |  |  |  | |  |  |
| Missing | 13 (2%) | 39 (1.5%) | 5 (1.5%) |  |  |  |  | |  |  |
| **Neck pain** |  |  |  | Chi2 = 0.6 | 1.0 | 0.009 (Negligible) |  | |  |  |
| Yes | 364 (58.1%) | 1500 (58%) | 198 (60.2%) |  |  |  |  | |  |  |
| No | 263 (41.9%) | 1088 (42%) | 131 (39.8%) |  |  |  |  | |  |  |
| Missing | 15 (2.3%) | 63 (2.4%) | 5 (1.5%) |  |  |  |  | |  |  |
| **Headaches** |  |  |  | Chi2 = 3.7 | 1.0 | 0.023 (Negligible) |  | |  |  |
| Yes | 256 (40.8%) | 993 (38.4%) | 142 (43.3%) |  |  |  |  | |  |  |
| No | 372 (59.2%) | 1596 (61.6%) | 186 (56.7%) |  |  |  |  | |  |  |
| Missing | 14 (2.2%) | 62 (2.3%) | 6 (1.8%) |  |  |  |  | |  |  |
| **Vertigo** |  |  |  | Chi2 = 9.5* | 0.154 | 0.036 (Negligible) |  | |  |  |
| Yes | 200 (32.3%) | 876 (34.1%) | 136 (42%) |  |  |  |  | |  |  |
| No | 419 (67.7%) | 1690 (65.9%) | 188 (58%) |  |  |  |  | |  |  |
| Missing | 23 (3.6%) | 85 (3.2%) | 10 (3%) |  |  |  |  | |  |  |
| **Number of treatment tested** |  |  |  | Chi2 = 36.0** | 0.003 | 0.032 (Negligible) |  | |  |  |
| 0 (none) | 76 (12%) | 392 (15%) | 63 (19%) |  |  |  | TW VS NE | | 0.002 | 0.039 (Negligible) |
| One | 83 (13.1%) | 441 (16.9%) | 48 (14.5%) |  |  |  | TI VS NE | | 1.0 | 0.021 (Negligible) |
| 2 to 4 | 175 (27.7%) | 776 (29.7%) | 92 (27.8%) |  |  |  | TW VS TI | | 0.175 | 0.03 (Negligible) |
| 5 and more | 148 (23.4%) | 453 (17.3%) | 67 (20.2%) |  |  |  |  | |  |  |
| several | 42 (6.6%) | 102 (3.9%) | 8 (2.4%) |  |  |  |  | |  |  |
| many | 108 (17.1%) | 453 (17.3%) | 53 (16%) |  |  |  |  | |  |  |
| Missing | 10 (1.6%) | 34 (1.3%) | 3 (0.9%) |  |  |  |  | |  |  |
| **Currently under psychiatric treatment** |  |  |  | Chi2 = 2.8 | 1.0 | 0.02 (Negligible) |  | |  |  |
| Yes | 135 (21.3%) | 481 (18.4%) | 63 (19%) |  |  |  |  | |  |  |
| No | 500 (78.7%) | 2139 (81.6%) | 268 (81%) |  |  |  |  | |  |  |
| Missing | 7 (1.1%) | 31 (1.2%) | 3 (0.9%) |  |  |  |  | |  |  |
| **Hearing aid user** |  |  |  | Chi2 = 24.9** | 0.013 | 0.034 (Negligible) |  | |  |  |
| right | 15 (2.4%) | 42 (1.6%) | 13 (4%) |  |  |  | TW VS NE | | 0.036 | 0.039 (Negligible) |
| left | 20 (3.2%) | 68 (2.6%) | 11 (3.4%) |  |  |  | TI VS NE | | 0.417 | 0.03 (Negligible) |
| both | 98 (15.6%) | 274 (10.5%) | 33 (10.3%) |  |  |  | TW VS TI | | 1.0 | 0.025 (Negligible) |
| none | 495 (78.8%) | 2220 (85.3%) | 264 (82.2%) |  |  |  |  | |  |  |
| Missing | 14 (2.2%) | 47 (1.8%) | 13 (3.9%) |  |  |  |  | |  |  |
