# Supplementary material for: Results of two cross-sectional database analyses regarding nap-induced modulations of tinnitus
Source: Sci Rep. 2024 Aug 29;14:20111. doi: 10.1038/s41598-024-70871-z (PMC11362562; doi:10.1038/s41598-024-70871-z)
Supplement: Supplementary file 2 — Supplementary Table 2. [file 41598_2024_70871_MOESM2_ESM.docx]

**Supplementary table 2**. Extended group comparison in the Tinnitus Hub survey database. *SD : Standard Deviation, TW : Tinnitus worsens after naps, NE : No effect of naps on tinnitus, TI : Tinnitus improves after naps,* *TMJ : temporo-mandibular joint, * : p < 0.05 before Holm correction, ** : p < 0.05 after Holm correction.*

|  | **Worsens N= 1404** | **No effect N= 4204** | **Improves N= 507** | **Statistic** | **p-Value** | **Effect size** |  | **Post-hoc test** | **Post-hoc effect size** |
| --- | --- | --- | --- | --- | --- | --- | --- | --- | --- |
| **Age (years)** |  |  |  | H = 17.6** | 0.009 | 0.003 (Negligible) |  |  |  |
| Mean (SD) | 54 (148) | 54.4 (13.6) | 51.7 (14.54) |  |  |  | TW VS NE | 1.0 | 0.03 (Negligible) |
| Median [Min Max] | 57 [14, 93] | 57 [6, 96] | 54 [15, 94] |  |  |  | TI VS NE | 0.002 | 0.197 (Negligible) |
| Missing | 0 (0%) | 0 (0%) | 0 (0%) |  |  |  | TW VS TI | 0.039 | 0.161 (Negligible) |
| **Tinnitus duration (months)** |  |  |  | H = 16.2** | 0.016 | 0.002 (Negligible) |  |  |  |
| Mean (SD) | 93.8 (100.93) | 106 (113.33) | 89 (105.63) |  |  |  | TW VS NE | 1.0 | 0.11 (Negligible) |
| Median [Min Max] | 52 [1, 371] | 59 [0, 371] | 42 [1, 371] |  |  |  | TI VS NE | 0.007 | 0.151 (Negligible) |
| Missing | 2 (0.1%) | 2 (0%) | 0 (0%) |  |  |  | TW VS TI | 0.796 | 0.047 (Negligible) |
| **Average loudness (0-10)** |  |  |  | H = 8.9* | 0.471 | 0.001 (Negligible) |  |  |  |
| Mean (SD) | 5.8 (2.33) | 5.6 (2.35) | 5.6 (2.13) |  |  |  |  |  |  |
| Median [Min Max] | 6 [1, 10] | 6 [0, 10] | 6 [0, 10] |  |  |  |  |  |  |
| Missing | 7 (0.5%) | 17 (0.4%) | 0 (0%) |  |  |  |  |  |  |
| **Loudness on good days (0-10)** |  |  |  | H = 9.3* | 0.404 | 0.001 (Negligible) |  |  |  |
| Mean (SD) | 3.6 (2.14) | 3.7 (2.24) | 3.4 (1.9) |  |  |  |  |  |  |
| Median [Min Max] | 3 [0, 10] | 3 [0, 10] | 3 [0, 10] |  |  |  |  |  |  |
| Missing | 13 (0.9%) | 39 (0.9%) | 3 (0.6%) |  |  |  |  |  |  |
| **Loudness on bad days (0-10)** |  |  |  | H = 8.6* | 0.525 | 0.001 (Negligible) |  |  |  |
| Mean (SD) | 6.6 (33) | 6.4 (2.87) | 6.6 (2.81) |  |  |  |  |  |  |
| Median [Min Max] | 7 [0, 10] | 7 [0, 10] | 7 [1, 10] |  |  |  |  |  |  |
| Missing | 30 (2.1%) | 73 (1.7%) | 7 (1.4%) |  |  |  |  |  |  |
| **Current loudness (0-10)** |  |  |  | H = 1.6 | 1.0 | -0.0 (Negligible) |  |  |  |
| Mean (SD) | 5.3 (2.51) | 5.2 (2.54) | 5.1 (2.34) |  |  |  |  |  |  |
| Median [Min Max] | 5 [0, 10] | 5 [0, 10] | 5 [0, 10] |  |  |  |  |  |  |
| Missing | 38 (2.7%) | 81 (1.9%) | 13 (2.6%) |  |  |  |  |  |  |
| **Average annoyance (0-10)** |  |  |  | H = 30.5** | p < 0.001 | 0.005 (Negligible) |  |  |  |
| Mean (SD) | 5.9 (2.99) | 5.4 (36) | 5.6 (2.94) |  |  |  | TW VS NE | p < 0.001 | 0.169 (Negligible) |
| Median [Min Max] | 6 [0, 10] | 5 [0, 10] | 5 [0, 10] |  |  |  | TI VS NE | 1.0 | 0.054 (Negligible) |
| Missing | 8 (0.6%) | 12 (0.3%) | 2 (0.4%) |  |  |  | TW VS TI | 1.0 | 0.118 (Negligible) |
| **Annoyance on good days (0-10)** |  |  |  | H = 3.3 | 1.0 | 0.0 (Negligible) |  |  |  |
| Mean (SD) | 3.6 (2.89) | 3.5 (2.95) | 3.3 (2.62) |  |  |  |  |  |  |
| Median [Min Max] | 3 [0, 10] | 3 [0, 10] | 3 [0, 10] |  |  |  |  |  |  |
| Missing | 20 (1.4%) | 36 (0.9%) | 4 (0.8%) |  |  |  |  |  |  |
| **Annoyance on bad days (0-10)** |  |  |  | H = 24.5** | p < 0.001 | 0.004 (Negligible) |  |  |  |
| Mean (SD) | 7 (3.2) | 6.5 (3.2) | 6.6 (3.19) |  |  |  | TW VS NE | p < 0.001 | 0.138 (Negligible) |
| Median [Min Max] | 8 [0, 10] | 7 [0, 10] | 7 [0, 10] |  |  |  | TI VS NE | 1.0 | 0.035 (Negligible) |
| Missing | 21 (1.5%) | 69 (1.6%) | 4 (0.8%) |  |  |  | TW VS TI | 0.914 | 0.103 (Negligible) |
| **Current annoyance (0-10)** |  |  |  | H = 7.2* | 0.952 | 0.001 (Negligible) |  |  |  |
| Mean (SD) | 5.3 (34) | 5.1 (39) | 5 (2.89) |  |  |  |  |  |  |
| Median [Min Max] | 5 [0, 10] | 5 [0, 10] | 5 [0, 10] |  |  |  |  |  |  |
| Missing | 27 (1.9%) | 76 (1.8%) | 11 (2.2%) |  |  |  |  |  |  |
| **% of time aware of tinnitus (0-100)** |  |  |  | H = 26.6** | p < 0.001 | 0.004 (Negligible) |  |  |  |
| Mean (SD) | 71.9 (26.17) | 67.7 (28.15) | 65.9 (26.81) |  |  |  | TW VS NE | p < 0.001 | 0.15 (Negligible) |
| Median [Min Max] | 76 [0, 100] | 75 [0, 100] | 70 [0, 100] |  |  |  | TI VS NE | 1.0 | 0.065 (Negligible) |
| Missing | 0 (0%) | 0 (0%) | 0 (0%) |  |  |  | TW VS TI | p < 0.001 | 0.226 (Small) |
| **Hearing loss grade (0 : none, 3 : severe)** |  |  |  | H = 3.1 | 1.0 | 0.0 (Negligible) |  |  |  |
| Mean (SD) | 1.2 (0.81) | 1.1 (0.8) | 1.1 (0.85) |  |  |  |  |  |  |
| Median [Min Max] | 1 [0, 3] | 1 [0, 3] | 1 [0, 3] |  |  |  |  |  |  |
| Missing | 0 (0%) | 0 (0%) | 0 (0%) |  |  |  |  |  |  |
| **Frequency of fleeting tinnitus (0 : never, 5 : daily)** |  |  |  | H = 60.0** | p < 0.001 | 0.009 (Negligible) |  |  |  |
| Mean (SD) | 2.9 (1.82) | 2.5 (1.91) | 2.8 (1.89) |  |  |  | TW VS NE | p < 0.001 | 0.228 (Small) |
| Median [Min Max] | 4 [0, 5] | 3 [0, 5] | 3 [0, 5] |  |  |  | TI VS NE | 0.022 | 0.161 (Negligible) |
| Missing | 0 (0%) | 0 (0%) | 0 (0%) |  |  |  | TW VS TI | 1.0 | 0.067 (Negligible) |
| **Gender** |  |  |  | Chi2 = 59.6** | p < 0.001 | 0.07 (Small) |  |  |  |
| Male | 832 (59.7%) | 2031 (48.6%) | 291 (57.9%) |  |  |  | TW VS NE | p < 0.001 | 0.092 (Negligible) |
| Female | 562 (40.3%) | 2151 (51.4%) | 212 (42.1%) |  |  |  | TI VS NE | 0.005 | 0.05 (Negligible) |
| Missing | 10 (0.7%) | 22 (0.5%) | 4 (0.8%) |  |  |  | TW VS TI | 1.0 | 0.008 (Negligible) |
| **Tinnitus sound : A pure tone** |  |  |  | Chi2 = 12.1* | 0.113 | 0.031 (Negligible) |  |  |  |
| No | 955 (68%) | 2651 (63.1%) | 336 (66.3%) |  |  |  |  |  |  |
| Yes | 449 (32%) | 1553 (36.9%) | 171 (33.7%) |  |  |  |  |  |  |
| Missing | 0 (0%) | 0 (0%) | 0 (0%) |  |  |  |  |  |  |
| **Tinnitus sound : A mixture of tones** |  |  |  | Chi2 = 21.7** | 0.001 | 0.042 (Negligible) |  |  |  |
| No | 904 (64.4%) | 2977 (70.8%) | 338 (66.7%) |  |  |  | TW VS NE | p < 0.001 | 0.057 (Negligible) |
| Yes | 500 (35.6%) | 1227 (29.2%) | 169 (33.3%) |  |  |  | TI VS NE | 1.0 | 0.024 (Negligible) |
| Missing | 0 (0%) | 0 (0%) | 0 (0%) |  |  |  | TW VS TI | 1.0 | 0.011 (Negligible) |
| **Tinnitus sound : A low buzzing** |  |  |  | Chi2 = 2.4 | 1.0 | 0.014 (Negligible) |  |  |  |
| No | 1213 (86.4%) | 3673 (87.4%) | 432 (85.2%) |  |  |  |  |  |  |
| Yes | 191 (13.6%) | 531 (12.6%) | 75 (14.8%) |  |  |  |  |  |  |
| Missing | 0 (0%) | 0 (0%) | 0 (0%) |  |  |  |  |  |  |
| **Tinnitus sound : A high buzzing** |  |  |  | Chi2 = 8.1* | 0.659 | 0.026 (Negligible) |  |  |  |
| No | 905 (64.5%) | 2748 (65.4%) | 299 (59%) |  |  |  |  |  |  |
| Yes | 499 (35.5%) | 1456 (34.6%) | 208 (41%) |  |  |  |  |  |  |
| Missing | 0 (0%) | 0 (0%) | 0 (0%) |  |  |  |  |  |  |
| **Tinnitus sound : electric/interference** |  |  |  | Chi2 = 17.3** | 0.01 | 0.038 (Negligible) |  |  |  |
| No | 1041 (74.1%) | 3337 (79.4%) | 402 (79.3%) |  |  |  | TW VS NE | 0.003 | 0.052 (Negligible) |
| Yes | 363 (25.9%) | 867 (20.6%) | 105 (20.7%) |  |  |  | TI VS NE | 1.0 | 0.0 (Negligible) |
| Missing | 0 (0%) | 0 (0%) | 0 (0%) |  |  |  | TW VS TI | 0.932 | 0.029 (Negligible) |
| **Tinnitus sound : A low rumbling** |  |  |  | Chi2 = 3.3 | 1.0 | 0.016 (Negligible) |  |  |  |
| No | 1325 (94.4%) | 4014 (95.5%) | 486 (95.9%) |  |  |  |  |  |  |
| Yes | 79 (5.6%) | 190 (4.5%) | 21 (4.1%) |  |  |  |  |  |  |
| Missing | 0 (0%) | 0 (0%) | 0 (0%) |  |  |  |  |  |  |
| **Tinnitus sound : A static noise** |  |  |  | Chi2 = 9.7* | 0.34 | 0.028 (Negligible) |  |  |  |
| No | 1124 (80.1%) | 3492 (83.1%) | 401 (79.1%) |  |  |  |  |  |  |
| Yes | 280 (19.9%) | 712 (16.9%) | 106 (20.9%) |  |  |  |  |  |  |
| Missing | 0 (0%) | 0 (0%) | 0 (0%) |  |  |  |  |  |  |
| **Tinnitus sound : clicking** |  |  |  | Chi2 = 7.4* | 0.882 | 0.025 (Negligible) |  |  |  |
| No | 1311 (93.4%) | 3983 (94.7%) | 468 (92.3%) |  |  |  |  |  |  |
| Yes | 93 (6.6%) | 221 (5.3%) | 39 (7.7%) |  |  |  |  |  |  |
| Missing | 0 (0%) | 0 (0%) | 0 (0%) |  |  |  |  |  |  |
| **Tinnitus sound : beeping (morse code)** |  |  |  | Chi2 = 1.6 | 1.0 | 0.011 (Negligible) |  |  |  |
| No | 1335 (95.1%) | 4027 (95.8%) | 482 (95.1%) |  |  |  |  |  |  |
| Yes | 69 (4.9%) | 177 (4.2%) | 25 (4.9%) |  |  |  |  |  |  |
| Missing | 0 (0%) | 0 (0%) | 0 (0%) |  |  |  |  |  |  |
| **Tinnitus sound : A pulsatile whooshing noise** |  |  |  | Chi2 = 2.0 | 1.0 | 0.013 (Negligible) |  |  |  |
| No | 1250 (89%) | 3786 (90.1%) | 449 (88.6%) |  |  |  |  |  |  |
| Yes | 154 (11%) | 418 (9.9%) | 58 (11.4%) |  |  |  |  |  |  |
| Missing | 0 (0%) | 0 (0%) | 0 (0%) |  |  |  |  |  |  |
| **Tinnitus sound : A non-pulsatile whooshing noise** |  |  |  | Chi2 = 6.1* | 1.0 | 0.022 (Negligible) |  |  |  |
| No | 1285 (91.5%) | 3915 (93.1%) | 461 (90.9%) |  |  |  |  |  |  |
| Yes | 119 (8.5%) | 289 (6.9%) | 46 (9.1%) |  |  |  |  |  |  |
| Missing | 0 (0%) | 0 (0%) | 0 (0%) |  |  |  |  |  |  |
| **Tinnitus sound : pulsatile sound** |  |  |  | Chi2 = 2.0 | 1.0 | 0.013 (Negligible) |  |  |  |
| No | 1287 (91.7%) | 3801 (90.4%) | 461 (90.9%) |  |  |  |  |  |  |
| Yes | 117 (8.3%) | 403 (9.6%) | 46 (9.1%) |  |  |  |  |  |  |
| Missing | 0 (0%) | 0 (0%) | 0 (0%) |  |  |  |  |  |  |
| **Tinnitus sound : other** |  |  |  | Chi2 = 3.8 | 1.0 | 0.018 (Negligible) |  |  |  |
| No | 1170 (83.3%) | 3499 (83.2%) | 439 (86.6%) |  |  |  |  |  |  |
| Yes | 234 (16.7%) | 705 (16.8%) | 68 (13.4%) |  |  |  |  |  |  |
| Missing | 0 (0%) | 0 (0%) | 0 (0%) |  |  |  |  |  |  |
| **Tinnitus cause : noise trauma** |  |  |  | Chi2 = 1.5 | 1.0 | 0.011 (Negligible) |  |  |  |
| No | 1169 (83.3%) | 3543 (84.3%) | 419 (82.6%) |  |  |  |  |  |  |
| Yes | 235 (16.7%) | 661 (15.7%) | 88 (17.4%) |  |  |  |  |  |  |
| Missing | 0 (0%) | 0 (0%) | 0 (0%) |  |  |  |  |  |  |
| **Tinnitus cause : hearing loss** |  |  |  | Chi2 = 4.8 | 1.0 | 0.02 (Negligible) |  |  |  |
| No | 1106 (78.8%) | 3393 (80.7%) | 421 (83%) |  |  |  |  |  |  |
| Yes | 298 (21.2%) | 811 (19.3%) | 86 (17%) |  |  |  |  |  |  |
| Missing | 0 (0%) | 0 (0%) | 0 (0%) |  |  |  |  |  |  |
| **Tinnitus cause : age-related hearing loss** |  |  |  | Chi2 = 6.4* | 1.0 | 0.023 (Negligible) |  |  |  |
| No | 1228 (87.5%) | 3741 (89%) | 434 (85.6%) |  |  |  |  |  |  |
| Yes | 176 (12.5%) | 463 (11%) | 73 (14.4%) |  |  |  |  |  |  |
| Missing | 0 (0%) | 0 (0%) | 0 (0%) |  |  |  |  |  |  |
| **Tinnitus cause : sudden hearing loss** |  |  |  | Chi2 = 6.2* | 1.0 | 0.023 (Negligible) |  |  |  |
| No | 1321 (94.1%) | 3946 (93.9%) | 462 (91.1%) |  |  |  |  |  |  |
| Yes | 83 (5.9%) | 258 (6.1%) | 45 (8.9%) |  |  |  |  |  |  |
| Missing | 0 (0%) | 0 (0%) | 0 (0%) |  |  |  |  |  |  |
| **Tinnitus cause : Meniere disease** |  |  |  | Chi2 = 6.6* | 1.0 | 0.023 (Negligible) |  |  |  |
| No | 1344 (95.7%) | 4016 (95.5%) | 472 (93.1%) |  |  |  |  |  |  |
| Yes | 60 (4.3%) | 188 (4.5%) | 35 (6.9%) |  |  |  |  |  |  |
| Missing | 0 (0%) | 0 (0%) | 0 (0%) |  |  |  |  |  |  |
| **Tinnitus cause : head or neck injury** |  |  |  | Chi2 = 26.0** | p < 0.001 | 0.046 (Negligible) |  |  |  |
| No | 1228 (87.5%) | 3859 (91.8%) | 448 (88.4%) |  |  |  | TW VS NE | p < 0.001 | 0.061 (Negligible) |
| Yes | 176 (12.5%) | 345 (8.2%) | 59 (11.6%) |  |  |  | TI VS NE | 0.525 | 0.032 (Negligible) |
| Missing | 0 (0%) | 0 (0%) | 0 (0%) |  |  |  | TW VS TI | 1.0 | 0.006 (Negligible) |
| **Tinnitus cause : barotrauma** |  |  |  | Chi2 = 5.1 | 1.0 | 0.02 (Negligible) |  |  |  |
| No | 1374 (97.9%) | 4073 (96.9%) | 497 (98%) |  |  |  |  |  |  |
| Yes | 30 (2.1%) | 131 (3.1%) | 10 (2%) |  |  |  |  |  |  |
| Missing | 0 (0%) | 0 (0%) | 0 (0%) |  |  |  |  |  |  |
| **Tinnitus cause : TMJ dysfunction** |  |  |  | Chi2 = 9.9* | 0.317 | 0.028 (Negligible) |  |  |  |
| No | 1242 (88.5%) | 3814 (90.7%) | 443 (87.4%) |  |  |  |  |  |  |
| Yes | 162 (11.5%) | 390 (9.3%) | 64 (12.6%) |  |  |  |  |  |  |
| Missing | 0 (0%) | 0 (0%) | 0 (0%) |  |  |  |  |  |  |
| **Tinnitus cause : psychological (stress, anxiety, depression)** |  |  |  | Chi2 = 60.1** | p < 0.001 | 0.07 (Small) |  |  |  |
| No | 1101 (78.4%) | 3593 (85.5%) | 382 (75.3%) |  |  |  | TW VS NE | p < 0.001 | 0.079 (Negligible) |
| Yes | 303 (21.6%) | 611 (14.5%) | 125 (24.7%) |  |  |  | TI VS NE | p < 0.001 | 0.075 (Negligible) |
| Missing | 0 (0%) | 0 (0%) | 0 (0%) |  |  |  | TW VS TI | 1.0 | 0.017 (Negligible) |
| **Tinnitus cause : ototoxicity** |  |  |  | Chi2 = 18.1** | 0.007 | 0.038 (Negligible) |  |  |  |
| No | 1212 (86.3%) | 3799 (90.4%) | 451 (89%) |  |  |  | TW VS NE | 0.001 | 0.054 (Negligible) |
| Yes | 192 (13.7%) | 405 (9.6%) | 56 (11%) |  |  |  | TI VS NE | 1.0 | 0.012 (Negligible) |
| Missing | 0 (0%) | 0 (0%) | 0 (0%) |  |  |  | TW VS TI | 1.0 | 0.018 (Negligible) |
| **Tinnitus cause : otosclerosis** |  |  |  | Chi2 = 0.4 | 1.0 | 0.006 (Negligible) |  |  |  |
| No | 1387 (98.8%) | 4150 (98.7%) | 499 (98.4%) |  |  |  |  |  |  |
| Yes | 17 (1.2%) | 54 (1.3%) | 8 (1.6%) |  |  |  |  |  |  |
| Missing | 0 (0%) | 0 (0%) | 0 (0%) |  |  |  |  |  |  |
| **Tinnitus cause : eustachian tube dysfunction** |  |  |  | Chi2 = 1.0 | 1.0 | 0.009 (Negligible) |  |  |  |
| No | 1312 (93.4%) | 3947 (93.9%) | 471 (92.9%) |  |  |  |  |  |  |
| Yes | 92 (6.6%) | 257 (6.1%) | 36 (7.1%) |  |  |  |  |  |  |
| Missing | 0 (0%) | 0 (0%) | 0 (0%) |  |  |  |  |  |  |
| **Tinnitus cause : dental treatment** |  |  |  | Chi2 = 2.2 | 1.0 | 0.013 (Negligible) |  |  |  |
| No | 1368 (97.4%) | 4069 (96.8%) | 488 (96.3%) |  |  |  |  |  |  |
| Yes | 36 (2.6%) | 135 (3.2%) | 19 (3.7%) |  |  |  |  |  |  |
| Missing | 0 (0%) | 0 (0%) | 0 (0%) |  |  |  |  |  |  |
| **Tinnitus cause : allergy** |  |  |  | Chi2 = 3.0 | 1.0 | 0.016 (Negligible) |  |  |  |
| No | 1365 (97.2%) | 4049 (96.3%) | 492 (97%) |  |  |  |  |  |  |
| Yes | 39 (2.8%) | 155 (3.7%) | 15 (3%) |  |  |  |  |  |  |
| Missing | 0 (0%) | 0 (0%) | 0 (0%) |  |  |  |  |  |  |
| **Tinnitus cause : ear wax procedure (syringing, candling...)** |  |  |  | Chi2 = 0.8 | 1.0 | 0.008 (Negligible) |  |  |  |
| No | 1366 (97.3%) | 4106 (97.7%) | 496 (97.8%) |  |  |  |  |  |  |
| Yes | 38 (2.7%) | 98 (2.3%) | 11 (2.2%) |  |  |  |  |  |  |
| Missing | 0 (0%) | 0 (0%) | 0 (0%) |  |  |  |  |  |  |
| **Tinnitus cause : metabolic (diabetes, thyroid, B12, hyperlipidaemia etc.)** |  |  |  | Chi2 = 13.4* | 0.064 | 0.033 (Negligible) |  |  |  |
| No | 1368 (97.4%) | 4069 (96.8%) | 477 (94.1%) |  |  |  |  |  |  |
| Yes | 36 (2.6%) | 135 (3.2%) | 30 (5.9%) |  |  |  |  |  |  |
| Missing | 0 (0%) | 0 (0%) | 0 (0%) |  |  |  |  |  |  |
| **Tinnitus cause : virus or infection** |  |  |  | Chi2 = 6.0 | 1.0 | 0.022 (Negligible) |  |  |  |
| No | 1186 (84.5%) | 3618 (86.1%) | 418 (82.4%) |  |  |  |  |  |  |
| Yes | 218 (15.5%) | 586 (13.9%) | 89 (17.6%) |  |  |  |  |  |  |
| Missing | 0 (0%) | 0 (0%) | 0 (0%) |  |  |  |  |  |  |
| **Tinnitus cause : ear wax build up** |  |  |  | Chi2 = 0.6 | 1.0 | 0.007 (Negligible) |  |  |  |
| No | 1366 (97.3%) | 4086 (97.2%) | 490 (96.6%) |  |  |  |  |  |  |
| Yes | 38 (2.7%) | 118 (2.8%) | 17 (3.4%) |  |  |  |  |  |  |
| Missing | 0 (0%) | 0 (0%) | 0 (0%) |  |  |  |  |  |  |
| **Tinnitus cause : unknown** |  |  |  | Chi2 = 18.8** | 0.005 | 0.039 (Negligible) |  |  |  |
| No | 72 (5.1%) | 122 (2.9%) | 26 (5.1%) |  |  |  | TW VS NE | 0.006 | 0.049 (Negligible) |
| Yes | 1332 (94.9%) | 4082 (97.1%) | 481 (94.9%) |  |  |  | TI VS NE | 0.455 | 0.033 (Negligible) |
| Missing | 0 (0%) | 0 (0%) | 0 (0%) |  |  |  | TW VS TI | 1.0 | 0.0 (Negligible) |
| **Fluctuations of tinnitus** |  |  |  | Chi2 = 257.3** | p < 0.001 | 0.084 (Small) |  |  |  |
| No fluctuations | 268 (19.1%) | 1512 (36%) | 80 (15.8%) |  |  |  | TW VS NE | p < 0.001 | 0.092 (Small) |
| Grows louder as day progresses | 703 (50.1%) | 1782 (42.4%) | 218 (43%) |  |  |  | TI VS NE | p < 0.001 | 0.084 (Small) |
| Grows quieter as day progresses | 122 (8.7%) | 193 (4.6%) | 36 (7.1%) |  |  |  | TW VS TI | p < 0.001 | 0.039 (Negligible) |
| Changes within the day or over days without any pattern | 311 (22.2%) | 717 (17.1%) | 173 (34.1%) |  |  |  |  |  |  |
| Missing | 0 (0%) | 0 (0%) | 0 (0%) |  |  |  |  |  |  |
| **Influence of stress over tinnitus** |  |  |  | Chi2 = 292.6** | p < 0.001 | 0.109 (Small) |  |  |  |
| Worsens | 1128 (80.3%) | 2439 (58%) | 409 (80.7%) |  |  |  | TW VS NE | p < 0.001 | 0.136 (Small) |
| No effect | 271 (19.3%) | 1746 (41.5%) | 95 (18.7%) |  |  |  | TI VS NE | p < 0.001 | 0.09 (Small) |
| Improves | 5 (0.4%) | 19 (0.5%) | 3 (0.6%) |  |  |  | TW VS TI | 1.0 | 0.007 (Negligible) |
| Missing | 0 (0%) | 0 (0%) | 0 (0%) |  |  |  |  |  |  |
| **Influence of anxiety over tinnitus** |  |  |  | Chi2 = 317.5** | p < 0.001 | 0.114 (Small) |  |  |  |
| Worsens | 1090 (77.6%) | 2272 (54%) | 394 (77.7%) |  |  |  | TW VS NE | p < 0.001 | 0.141 (Small) |
| No effect | 309 (22%) | 1910 (45.4%) | 107 (21.1%) |  |  |  | TI VS NE | p < 0.001 | 0.095 (Small) |
| Improves | 5 (0.4%) | 22 (0.5%) | 6 (1.2%) |  |  |  | TW VS TI | 1.0 | 0.019 (Negligible) |
| Missing | 0 (0%) | 0 (0%) | 0 (0%) |  |  |  |  |  |  |
| **Influence of a good night sleep over tinnitus** |  |  |  | Chi2 = 1373.5** | p < 0.001 | 0.237 (Medium) |  |  |  |
| Worsens | 476 (33.9%) | 272 (6.5%) | 24 (4.7%) |  |  |  | TW VS NE | p < 0.001 | 0.257 (Medium) |
| No effect | 430 (30.6%) | 2665 (63.4%) | 67 (13.2%) |  |  |  | TI VS NE | p < 0.001 | 0.21 (Medium) |
| Improves | 498 (35.5%) | 1267 (30.1%) | 416 (82.1%) |  |  |  | TW VS TI | p < 0.001 | 0.165 (Small) |
| Missing | 0 (0%) | 0 (0%) | 0 (0%) |  |  |  |  |  |  |
| **Influence of poor sleep over tinnitus** |  |  |  | Chi2 = 694.5** | p < 0.001 | 0.169 (Medium) |  |  |  |
| Worsens | 1134 (80.8%) | 1844 (43.9%) | 379 (74.8%) |  |  |  | TW VS NE | p < 0.001 | 0.22 (Medium) |
| No effect | 250 (17.8%) | 2320 (55.2%) | 118 (23.3%) |  |  |  | TI VS NE | p < 0.001 | 0.123 (Small) |
| Improves | 20 (1.4%) | 40 (1%) | 10 (2%) |  |  |  | TW VS TI | 0.706 | 0.026 (Negligible) |
| Missing | 0 (0%) | 0 (0%) | 0 (0%) |  |  |  |  |  |  |
| **Some sounds can worsen tinnitus** |  |  |  | Chi2 = 20.0** | 0.003 | 0.04 (Negligible) |  |  |  |
| No | 663 (47.2%) | 2268 (53.9%) | 254 (50.1%) |  |  |  | TW VS NE | p < 0.001 | 0.055 (Negligible) |
| Yes | 741 (52.8%) | 1936 (46.1%) | 253 (49.9%) |  |  |  | TI VS NE | 1.0 | 0.02 (Negligible) |
| Missing | 0 (0%) | 0 (0%) | 0 (0%) |  |  |  | TW VS TI | 1.0 | 0.014 (Negligible) |
| **Some sounds can reduce tinnitus** |  |  |  | Chi2 = 36.6** | p < 0.001 | 0.055 (Negligible) |  |  |  |
| No | 426 (30.3%) | 1002 (23.8%) | 167 (32.9%) |  |  |  | TW VS NE | p < 0.001 | 0.062 (Negligible) |
| Yes | 978 (69.7%) | 3202 (76.2%) | 340 (67.1%) |  |  |  | TI VS NE | p < 0.001 | 0.057 (Negligible) |
| Missing | 0 (0%) | 0 (0%) | 0 (0%) |  |  |  | TW VS TI | 1.0 | 0.013 (Negligible) |
| **Tinnitus sound masking** |  |  |  | Chi2 = 128.7** | p < 0.001 | 0.046 (Small) |  |  |  |
| No masking | 101 (7.2%) | 410 (9.8%) | 69 (13.6%) |  |  |  | TW VS NE | p < 0.001 | 0.058 (Small) |
| Only a small selection of specific sounds | 162 (11.5%) | 357 (8.5%) | 63 (12.4%) |  |  |  | TI VS NE | p < 0.001 | 0.033 (Negligible) |
| Shower / water noises | 287 (20.4%) | 485 (11.5%) | 68 (13.4%) |  |  |  | TW VS TI | p < 0.001 | 0.033 (Negligible) |
| TV, Music or general background noise | 334 (23.8%) | 1291 (30.7%) | 135 (26.6%) |  |  |  |  |  |  |
| White noise or special masking noises | 197 (14%) | 555 (13.2%) | 80 (15.8%) |  |  |  |  |  |  |
| Masked by nearly all sounds | 323 (23%) | 1106 (26.3%) | 92 (18.1%) |  |  |  |  |  |  |
| Missing | 0 (0%) | 0 (0%) | 0 (0%) |  |  |  |  |  |  |
| **Influence of intense workout over tinnitus** |  |  |  | Chi2 = 252.2** | p < 0.001 | 0.102 (Small) |  |  |  |
| Worsens | 424 (30.2%) | 645 (15.3%) | 126 (24.9%) |  |  |  | TW VS NE | p < 0.001 | 0.121 (Small) |
| No effect | 847 (60.3%) | 3282 (78.1%) | 295 (58.2%) |  |  |  | TI VS NE | p < 0.001 | 0.096 (Small) |
| Improves | 133 (9.5%) | 277 (6.6%) | 86 (17%) |  |  |  | TW VS TI | p < 0.001 | 0.043 (Negligible) |
| Missing | 0 (0%) | 0 (0%) | 0 (0%) |  |  |  |  |  |  |
| **Influence of moderate workout over tinnitus** |  |  |  | Chi2 = 326.7** | p < 0.001 | 0.116 (Small) |  |  |  |
| Worsens | 423 (30.1%) | 584 (13.9%) | 117 (23.1%) |  |  |  | TW VS NE | p < 0.001 | 0.135 (Small) |
| No effect | 801 (57.1%) | 3219 (76.6%) | 267 (52.7%) |  |  |  | TI VS NE | p < 0.001 | 0.11 (Small) |
| Improves | 180 (12.8%) | 401 (9.5%) | 123 (24.3%) |  |  |  | TW VS TI | p < 0.001 | 0.056 (Negligible) |
| Missing | 0 (0%) | 0 (0%) | 0 (0%) |  |  |  |  |  |  |
| **Influence of light exercise over tinnitus** |  |  |  | Chi2 = 320.8** | p < 0.001 | 0.115 (Small) |  |  |  |
| Worsens | 231 (16.5%) | 284 (6.8%) | 59 (11.6%) |  |  |  | TW VS NE | p < 0.001 | 0.113 (Small) |
| No effect | 937 (66.7%) | 3424 (81.4%) | 278 (54.8%) |  |  |  | TI VS NE | p < 0.001 | 0.13 (Small) |
| Improves | 236 (16.8%) | 496 (11.8%) | 170 (33.5%) |  |  |  | TW VS TI | p < 0.001 | 0.072 (Small) |
| Missing | 0 (0%) | 0 (0%) | 0 (0%) |  |  |  |  |  |  |
| **Somatosensory change : pressing the jaw on the side** |  |  |  | Chi2 = 48.3** | p < 0.001 | 0.063 (Negligible) |  |  |  |
| No | 1121 (79.8%) | 3670 (87.3%) | 422 (83.2%) |  |  |  | TW VS NE | p < 0.001 | 0.087 (Negligible) |
| Yes | 283 (20.2%) | 534 (12.7%) | 85 (16.8%) |  |  |  | TI VS NE | 0.564 | 0.032 (Negligible) |
| Missing | 0 (0%) | 0 (0%) | 0 (0%) |  |  |  | TW VS TI | 1.0 | 0.02 (Negligible) |
| **Somatosensory change : pushing jaw backwards** |  |  |  | Chi2 = 24.2** | p < 0.001 | 0.044 (Negligible) |  |  |  |
| No | 1151 (82%) | 3667 (87.2%) | 439 (86.6%) |  |  |  | TW VS NE | p < 0.001 | 0.062 (Negligible) |
| Yes | 253 (18%) | 537 (12.8%) | 68 (13.4%) |  |  |  | TI VS NE | 1.0 | 0.004 (Negligible) |
| Missing | 0 (0%) | 0 (0%) | 0 (0%) |  |  |  | TW VS TI | 0.837 | 0.03 (Negligible) |
| **Somatosensory change : Pushing the jaw outwards rapidly** |  |  |  | Chi2 = 37.8** | p < 0.001 | 0.056 (Negligible) |  |  |  |
| No | 1022 (72.8%) | 3385 (80.5%) | 404 (79.7%) |  |  |  | TW VS NE | p < 0.001 | 0.078 (Negligible) |
| Yes | 382 (27.2%) | 819 (19.5%) | 103 (20.3%) |  |  |  | TI VS NE | 1.0 | 0.005 (Negligible) |
| Missing | 0 (0%) | 0 (0%) | 0 (0%) |  |  |  | TW VS TI | 0.131 | 0.038 (Negligible) |
| **Somatosensory change : Pushing your hand against your forehead while resisting with the neck muscles** |  |  |  | Chi2 = 22.9** | p < 0.001 | 0.043 (Negligible) |  |  |  |
| No | 1094 (77.9%) | 3513 (83.6%) | 418 (82.4%) |  |  |  | TW VS NE | p < 0.001 | 0.061 (Negligible) |
| Yes | 310 (22.1%) | 691 (16.4%) | 89 (17.6%) |  |  |  | TI VS NE | 1.0 | 0.007 (Negligible) |
| Missing | 0 (0%) | 0 (0%) | 0 (0%) |  |  |  | TW VS TI | 1.0 | 0.027 (Negligible) |
| **Somatosensory change : clenching teeth** |  |  |  | Chi2 = 32.1** | p < 0.001 | 0.051 (Negligible) |  |  |  |
| No | 1006 (71.7%) | 3313 (78.8%) | 377 (74.4%) |  |  |  | TW VS NE | p < 0.001 | 0.07 (Negligible) |
| Yes | 398 (28.3%) | 891 (21.2%) | 130 (25.6%) |  |  |  | TI VS NE | 0.932 | 0.029 (Negligible) |
| Missing | 0 (0%) | 0 (0%) | 0 (0%) |  |  |  | TW VS TI | 1.0 | 0.014 (Negligible) |
| **Somatosensory change : Tilting your head backwards** |  |  |  | Chi2 = 34.7** | p < 0.001 | 0.053 (Negligible) |  |  |  |
| No | 1132 (80.6%) | 3656 (87%) | 424 (83.6%) |  |  |  | TW VS NE | p < 0.001 | 0.074 (Negligible) |
| Yes | 272 (19.4%) | 548 (13%) | 83 (16.4%) |  |  |  | TI VS NE | 1.0 | 0.026 (Negligible) |
| Missing | 0 (0%) | 0 (0%) | 0 (0%) |  |  |  | TW VS TI | 1.0 | 0.018 (Negligible) |
| **Somatosensory change : no change with any of these actions** |  |  |  | Chi2 = 62.4** | p < 0.001 | 0.071 (Small) |  |  |  |
| No | 734 (52.3%) | 1703 (40.5%) | 241 (47.5%) |  |  |  | TW VS NE | p < 0.001 | 0.098 (Negligible) |
| Yes | 670 (47.7%) | 2501 (59.5%) | 266 (52.5%) |  |  |  | TI VS NE | 0.132 | 0.038 (Negligible) |
| Missing | 0 (0%) | 0 (0%) | 0 (0%) |  |  |  | TW VS TI | 1.0 | 0.023 (Negligible) |
| **Jaw : my jaw sometimes feels painful** |  |  |  | Chi2 = 1.7 | 1.0 | 0.012 (Negligible) |  |  |  |
| No | 1238 (88.2%) | 3759 (89.4%) | 450 (88.8%) |  |  |  |  |  |  |
| Yes | 166 (11.8%) | 445 (10.6%) | 57 (11.2%) |  |  |  |  |  |  |
| Missing | 0 (0%) | 0 (0%) | 0 (0%) |  |  |  |  |  |  |
| **Jaw : I struggle to fully move my jaw** |  |  |  | Chi2 = 4.3 | 1.0 | 0.019 (Negligible) |  |  |  |
| No | 1358 (96.7%) | 4107 (97.7%) | 496 (97.8%) |  |  |  |  |  |  |
| Yes | 46 (3.3%) | 97 (2.3%) | 11 (2.2%) |  |  |  |  |  |  |
| Missing | 0 (0%) | 0 (0%) | 0 (0%) |  |  |  |  |  |  |
| **Jaw : I struggle to fully move my jaw** |  |  |  | Chi2 = 13.4* | 0.064 | 0.033 (Negligible) |  |  |  |
| No | 1277 (91%) | 3877 (92.2%) | 444 (87.6%) |  |  |  |  |  |  |
| Yes | 127 (9%) | 327 (7.8%) | 63 (12.4%) |  |  |  |  |  |  |
| Missing | 0 (0%) | 0 (0%) | 0 (0%) |  |  |  |  |  |  |
| **Jaw : muscles around my jaw feel tight or tense** |  |  |  | Chi2 = 6.3* | 1.0 | 0.023 (Negligible) |  |  |  |
| No | 1171 (83.4%) | 3612 (85.9%) | 424 (83.6%) |  |  |  |  |  |  |
| Yes | 233 (16.6%) | 592 (14.1%) | 83 (16.4%) |  |  |  |  |  |  |
| Missing | 0 (0%) | 0 (0%) | 0 (0%) |  |  |  |  |  |  |
| **Jaw : I have several popping and clicking noises in my jaw** |  |  |  | Chi2 = 12.7* | 0.087 | 0.032 (Negligible) |  |  |  |
| No | 1104 (78.6%) | 3479 (82.8%) | 406 (80.1%) |  |  |  |  |  |  |
| Yes | 300 (21.4%) | 725 (17.2%) | 101 (19.9%) |  |  |  |  |  |  |
| Missing | 0 (0%) | 0 (0%) | 0 (0%) |  |  |  |  |  |  |
| **Jaw : I have been diagnosed with TMJ dysfunction** |  |  |  | Chi2 = 3.2 | 1.0 | 0.016 (Negligible) |  |  |  |
| No | 1296 (92.3%) | 3925 (93.4%) | 465 (91.7%) |  |  |  |  |  |  |
| Yes | 108 (7.7%) | 279 (6.6%) | 42 (8.3%) |  |  |  |  |  |  |
| Missing | 0 (0%) | 0 (0%) | 0 (0%) |  |  |  |  |  |  |
| **Jaw : No real issues that I am aware of** |  |  |  | Chi2 = 9.6* | 0.358 | 0.028 (Negligible) |  |  |  |
| No | 530 (37.7%) | 1415 (33.7%) | 191 (37.7%) |  |  |  |  |  |  |
| Yes | 874 (62.3%) | 2789 (66.3%) | 316 (62.3%) |  |  |  |  |  |  |
| Missing | 0 (0%) | 0 (0%) | 0 (0%) |  |  |  |  |  |  |
| **Bruxism : I grind my teeth during my sleep** |  |  |  | Chi2 = 1.0 | 1.0 | 0.009 (Negligible) |  |  |  |
| No | 960 (68.4%) | 2898 (68.9%) | 339 (66.9%) |  |  |  |  |  |  |
| Yes | 444 (31.6%) | 1306 (31.1%) | 168 (33.1%) |  |  |  |  |  |  |
| Missing | 0 (0%) | 0 (0%) | 0 (0%) |  |  |  |  |  |  |
| **Bruxism : I often clench my teeth without realizing it** |  |  |  | Chi2 = 7.8* | 0.751 | 0.025 (Negligible) |  |  |  |
| No | 1027 (73.1%) | 3105 (73.9%) | 345 (68%) |  |  |  |  |  |  |
| Yes | 377 (26.9%) | 1099 (26.1%) | 162 (32%) |  |  |  |  |  |  |
| Missing | 0 (0%) | 0 (0%) | 0 (0%) |  |  |  |  |  |  |
| **Neck stiffness : after certain physical activity** |  |  |  | Chi2 = 2.9 | 1.0 | 0.015 (Negligible) |  |  |  |
| No | 1177 (83.8%) | 3569 (84.9%) | 417 (82.2%) |  |  |  |  |  |  |
| Yes | 227 (16.2%) | 635 (15.1%) | 90 (17.8%) |  |  |  |  |  |  |
| Missing | 0 (0%) | 0 (0%) | 0 (0%) |  |  |  |  |  |  |
| **Neck stiffness : from bad posture** |  |  |  | Chi2 = 11.2* | 0.175 | 0.03 (Negligible) |  |  |  |
| No | 1062 (75.6%) | 3332 (79.3%) | 380 (75%) |  |  |  |  |  |  |
| Yes | 342 (24.4%) | 872 (20.7%) | 127 (25%) |  |  |  |  |  |  |
| Missing | 0 (0%) | 0 (0%) | 0 (0%) |  |  |  |  |  |  |
| **Neck stiffness : I have an associated medical condition** |  |  |  | Chi2 = 0.2 | 1.0 | 0.004 (Negligible) |  |  |  |
| No | 1259 (89.7%) | 3753 (89.3%) | 454 (89.5%) |  |  |  |  |  |  |
| Yes | 145 (10.3%) | 451 (10.7%) | 53 (10.5%) |  |  |  |  |  |  |
| Missing | 0 (0%) | 0 (0%) | 0 (0%) |  |  |  |  |  |  |
| **Neck stiffness : from lying in bed / sleeping** |  |  |  | Chi2 = 7.4* | 0.876 | 0.025 (Negligible) |  |  |  |
| No | 1077 (76.7%) | 3364 (80%) | 395 (77.9%) |  |  |  |  |  |  |
| Yes | 327 (23.3%) | 840 (20%) | 112 (22.1%) |  |  |  |  |  |  |
| Missing | 0 (0%) | 0 (0%) | 0 (0%) |  |  |  |  |  |  |
| **Neck stiffness : my neck movement is restricted due to stiffness** |  |  |  | Chi2 = 4.1 | 1.0 | 0.018 (Negligible) |  |  |  |
| No | 1182 (84.2%) | 3523 (83.8%) | 408 (80.5%) |  |  |  |  |  |  |
| Yes | 222 (15.8%) | 681 (16.2%) | 99 (19.5%) |  |  |  |  |  |  |
| Missing | 0 (0%) | 0 (0%) | 0 (0%) |  |  |  |  |  |  |
| **Neck stiffness : No more than I believe is normal** |  |  |  | Chi2 = 10.1* | 0.293 | 0.029 (Negligible) |  |  |  |
| No | 797 (56.8%) | 2190 (52.1%) | 281 (55.4%) |  |  |  |  |  |  |
| Yes | 607 (43.2%) | 2014 (47.9%) | 226 (44.6%) |  |  |  |  |  |  |
| Missing | 0 (0%) | 0 (0%) | 0 (0%) |  |  |  |  |  |  |
| **Headaches** |  |  |  | Chi2 = 12.4* | 0.101 | 0.032 (Negligible) |  |  |  |
| No | 821 (58.5%) | 2667 (63.4%) | 302 (59.6%) |  |  |  |  |  |  |
| Yes | 583 (41.5%) | 1537 (36.6%) | 205 (40.4%) |  |  |  |  |  |  |
| Missing | 0 (0%) | 0 (0%) | 0 (0%) |  |  |  |  |  |  |
| **Headaches coming from the neck** |  |  |  | Chi2 = 2.5 | 1.0 | 0.014 (Negligible) |  |  |  |
| No | 1100 (78.3%) | 3369 (80.1%) | 398 (78.5%) |  |  |  |  |  |  |
| Yes | 304 (21.7%) | 835 (19.9%) | 109 (21.5%) |  |  |  |  |  |  |
| Missing | 0 (0%) | 0 (0%) | 0 (0%) |  |  |  |  |  |  |
| **Headaches coming from the jaw** |  |  |  | Chi2 = 8.8* | 0.497 | 0.027 (Negligible) |  |  |  |
| No | 1281 (91.2%) | 3908 (93%) | 456 (89.9%) |  |  |  |  |  |  |
| Yes | 123 (8.8%) | 296 (7%) | 51 (10.1%) |  |  |  |  |  |  |
| Missing | 0 (0%) | 0 (0%) | 0 (0%) |  |  |  |  |  |  |
| **Ear fullness : after activity mainly** |  |  |  | Chi2 = 28.4** | p < 0.001 | 0.048 (Negligible) |  |  |  |
| No | 1309 (93.2%) | 4058 (96.5%) | 480 (94.7%) |  |  |  | TW VS NE | p < 0.001 | 0.066 (Negligible) |
| Yes | 95 (6.8%) | 146 (3.5%) | 27 (5.3%) |  |  |  | TI VS NE | 1.0 | 0.025 (Negligible) |
| Missing | 0 (0%) | 0 (0%) | 0 (0%) |  |  |  | TW VS TI | 1.0 | 0.013 (Negligible) |
| **Ear fullness : after a bad sleep** |  |  |  | Chi2 = 72.7** | p < 0.001 | 0.077 (Small) |  |  |  |
| No | 1250 (89%) | 3994 (95%) | 452 (89.2%) |  |  |  | TW VS NE | p < 0.001 | 0.1 (Small) |
| Yes | 154 (11%) | 210 (5%) | 55 (10.8%) |  |  |  | TI VS NE | p < 0.001 | 0.068 (Negligible) |
| Missing | 0 (0%) | 0 (0%) | 0 (0%) |  |  |  | TW VS TI | 1.0 | 0.0 (Negligible) |
| **Ear fullness : after listening to some sounds or being exposed to noise** |  |  |  | Chi2 = 12.4* | 0.1 | 0.032 (Negligible) |  |  |  |
| No | 1214 (86.5%) | 3760 (89.4%) | 436 (86%) |  |  |  |  |  |  |
| Yes | 190 (13.5%) | 444 (10.6%) | 71 (14%) |  |  |  |  |  |  |
| Missing | 0 (0%) | 0 (0%) | 0 (0%) |  |  |  |  |  |  |
| **Ear fullness : after working at a computer or desk** |  |  |  | Chi2 = 22.5** | p < 0.001 | 0.043 (Negligible) |  |  |  |
| No | 1355 (96.5%) | 4101 (97.5%) | 476 (93.9%) |  |  |  | TW VS NE | 1.0 | 0.025 (Negligible) |
| Yes | 49 (3.5%) | 103 (2.5%) | 31 (6.1%) |  |  |  | TI VS NE | p < 0.001 | 0.058 (Negligible) |
| Missing | 0 (0%) | 0 (0%) | 0 (0%) |  |  |  | TW VS TI | 0.706 | 0.031 (Negligible) |
| **Ear fullness : after periods of stress / anxiety** |  |  |  | Chi2 = 85.3** | p < 0.001 | 0.084 (Small) |  |  |  |
| No | 1129 (80.4%) | 3700 (88%) | 386 (76.1%) |  |  |  | TW VS NE | p < 0.001 | 0.091 (Negligible) |
| Yes | 275 (19.6%) | 504 (12%) | 121 (23.9%) |  |  |  | TI VS NE | p < 0.001 | 0.094 (Negligible) |
| Missing | 0 (0%) | 0 (0%) | 0 (0%) |  |  |  | TW VS TI | 1.0 | 0.025 (Negligible) |
| **Ear fullness : yes but no identified cause** |  |  |  | Chi2 = 2.6 | 1.0 | 0.015 (Negligible) |  |  |  |
| No | 882 (62.8%) | 2549 (60.6%) | 318 (62.7%) |  |  |  |  |  |  |
| Yes | 522 (37.2%) | 1655 (39.4%) | 189 (37.3%) |  |  |  |  |  |  |
| Missing | 0 (0%) | 0 (0%) | 0 (0%) |  |  |  |  |  |  |
| **No ear fullness** |  |  |  | Chi2 = 42.3** | p < 0.001 | 0.059 (Negligible) |  |  |  |
| No | 907 (64.6%) | 2386 (56.8%) | 343 (67.7%) |  |  |  | TW VS NE | p < 0.001 | 0.066 (Negligible) |
| Yes | 497 (35.4%) | 1818 (43.2%) | 164 (32.3%) |  |  |  | TI VS NE | p < 0.001 | 0.059 (Negligible) |
| Missing | 0 (0%) | 0 (0%) | 0 (0%) |  |  |  | TW VS TI | 1.0 | 0.015 (Negligible) |
|  |  |  |  |  |  |  |  |  |  |
